# Transfusion requirements in septic shock patients: a randomized controlled trial

**DOI:** 10.1186/cc13302

**Published:** 2014-03-17

**Authors:** F Bergamin, J Almeida, C Park, E Osawa, J Silva, F Galas, D Nagaoka, J Fukushima, S Vieira, L Candido, CO Oshiro, JL Vincent, L Hajjar

**Affiliations:** 1 Instituto do Cancer do Estado de São Paulo, Brazil; 2 Erasme Hospital, Université libre de Bruxelles, Brussels, Belgium

## Introduction

Perioperative red blood cell transfusion is commonly used to address anemia, an independent risk factor for morbidity and mortality in critically ill patients [1]; however, evidence regarding optimal blood transfusion practice in septic shock is lacking. The aim of this study was to define which is the best transfusion strategy in septic shock patients regarding 28-day mortality and clinical outcomes: restrictive or liberal.

## Methods

The Transfusion Requirements After Cardiac Surgery (TRACS) study is a prospective, randomized, controlled clinical noninferiority trial conducted between February 2009 and February 2010 in an ICU at a university hospital cardiac surgery referral center in Brazil. Consecutive adult patients (*n *= 502) who underwent cardiac surgery with cardiopulmonary bypass were eligible; analysis was by intention to treat.

This is a randomized controlled parallel-group trial, which included 300 patients admitted to a cancer ICU with diagnosis of septic shock. Patients were randomly assigned to a liberal strategy of blood transfusion (to maintain hemoglobin >9 g/dl) or to a restrictive strategy (hemoglobin >7 g/dl). Mortality in 28 days was the main outcome. Secondary outcomes were clinical complications days free of organ dysfunction, ICU and hospital length of stay, adverse effects of transfusion and 60-day mortality.

## Results

A total of 136 patients were included in the first part of the trial. Mean age was 62 ± 14 years, SAPS 3 at admission was 65 ± 15 and all patients had the diagnosis of solid neoplasm. Sixty-three patients (46.3%) were included in the liberal strategy and 73 patients (53.7%) in the restrictive strategy. Occurrence of 28-day mortality was similar between groups (54% in liberal group vs. 56.2% in restrictive group; *P *= 0.395).

## Conclusion

Among cancer patients with septic shock, the use of a restrictive perioperative transfusion strategy compared with a more liberal strategy resulted in similar rates of 28-day-mortality.
